# Classifying primary central nervous system lymphoma from glioblastoma using deep learning and radiomics based machine learning approach - a systematic review and meta-analysis

**DOI:** 10.3389/fonc.2022.884173

**Published:** 2022-10-03

**Authors:** Amrita Guha, Jayant S. Goda, Archya Dasgupta, Abhishek Mahajan, Soutik Halder, Jeetendra Gawde, Sanjay Talole

**Affiliations:** ^1^ Department of Radio Diagnosis, Tata Memorial Centre, Homi Bhaba National Institute, Mumbai, India; ^2^ Department of Radiation Oncology, Tata Memorial Centre, Homi Bhaba National Institute, Mumbai, India; ^3^ Department of Biostatistics, Tata Memorial Centre, Homi Bhaba National Institute, Mumbai, India

**Keywords:** machine learning, deep learning, predictive analytics, primary central nervous system (CNS) lymphoma, glioblastoma, magnetic resonance imaging, metaanalysis, systematic review

## Abstract

**Background:**

Glioblastoma (GBM) and primary central nervous system lymphoma (PCNSL) are common in elderly yet difficult to differentiate on MRI. Their management and prognosis are quite different. Recent surge of interest in predictive analytics, using machine learning (ML) from radiomic features and deep learning (DL) for diagnosing, predicting response and prognosticating disease has evinced interest among radiologists and clinicians. The objective of this systematic review and meta-analysis was to evaluate the deep learning & ML algorithms in classifying PCNSL from GBM.

**Methods:**

The authors performed a systematic review of the literature from MEDLINE, EMBASE and the Cochrane central trials register for the search strategy in accordance with PRISMA guidelines to select and evaluate studies that included themes of ML, DL, AI, GBM, PCNSL. All studies reporting on ML algorithms or DL that for differentiating PCNSL from GBM on MR imaging were included. These studies were further narrowed down to focus on works published between 2018 and 2021. Two researchers independently conducted the literature screening, database extraction and risk bias assessment. The extracted data was synthesised and analysed by forest plots. Outcomes assessed were test characteristics such as accuracy, sensitivity, specificity and balanced accuracy.

**Results:**

Ten articles meeting the eligibility criteria were identified addressing use of ML and DL in training and validation classifiers to distinguish PCNSL from GBM on MR imaging. The total sample size was 1311 in the included studies. ML approach was used in 6 studies while DL in 4 studies. The lowest reported sensitivity was 80%, while the highest reported sensitivity was 99% in studies in which ML and DL was directly compared with the gold standard histopathology. The lowest reported specificity was 87% while the highest reported specificity was 100%. The highest reported balanced accuracy was 100% and the lowest was 84%.

**Conclusions:**

Extensive search of the database revealed a limited number of studies that have applied ML or DL to differentiate PCNSL from GBM. Of the currently published studies, Both DL & ML algorithms have demonstrated encouraging results and certainly have the potential to aid neurooncologists in taking preoperative decisions in the future leading to not only reduction in morbidities but also be cost effective.

## Introduction

Primary Central Nervous System Lymphomas (PCNSL) and Glioblastomas(GBM) are tumours of the adults and elderly, however, they are distinct entities in terms of their cell of origin, incidence, natural history, treatment protocols and prognosis (1). Even though these tumours are different, they appear radiologically appear similar on Magnetic resonance imaging(MRI) with only a few discerning features (2). Although there are a few semantic MR imaging features that help the radiologist to differentiate PCNSL from GBM (3), these features are subjective and dependant on the expertise and the experience of the radiologist with a resultant dependence on the gold standard histopathology of the tumour specimen (4). Certain special MRI sequences such as Diffusion Weighted Imaging (DWI), MR spectroscopy (MRS) may complement the semantic features (5), and could be useful in differentiating the two tumours but these special MR protocols are resource intense and their use is limited due to lack of widespread availability and associated cost escalations have practise implications in high throughput cancer centres.

Tumor radiomics based on texture feature analysis of MR images represents an abstract mathematical quantitative approach whereby multiple individual imaging features not easily discerned by the naked eye are processed by means of sophisticated algorithms to reveal quantifiable indices (6). Radiomics maximizes the number of quantitative image features from digital images and as a result, can overcome intratumoral heterogeneities in both the molecular and histopathological assessment of various tumour histologies using measurable values that contribute to tumor diagnosis, pre-surgical grading, response to treatment, prognostication of cancers and predicting gene mutation. Moreover, with quantified analyses of images, it has also been incorporated with various novel computer technologies, such as machine learning and deep learning algorithms like deep convolutional neural network (dCNN) (7–15)

Even before deep learning methods were available, majority of ML based radiology studies used texture features extracted from manually segmented tumour images followed by application of conventional ML tools such as random forests and support vector machines (15–17) The advent of advanced computational methods like deep learning algorithms brought a paradigm shift in the image based classification of tumours and their biology (18). The development of the convolutional neural network (CNN), that comprises of convolution and pooling layers, has led to automation in identifying relevant image features for various classification tasks (19).

Although, various ML tools like random forest or support vector machine models and DL algorithms like CNN have been used to classify PCNSL from GBM, the results have been heterogeneous in terms of the specificity, sensitivity and accuracy of the various computational methods in differentiating these tumours precluding their use in clinical practice. Therefore, there remains a need for systematic and thorough review of all the existing literature that have looked into the classification aspect PCNSL vs GBM by various ML and DL tools.

Thus, the purpose of this systematic review and metanalysis was to estimate the diagnostic accuracy of ML-based radiomics and DL models in classifying PCNSL and GBM in an endeavour to eventually help neurooncologists in their management decisions upfront. In addition, we evaluated different combinations of selection methods and classifiers, trying to make comparison of models’ performances.

## Methods

### Literature review

**Figure 1:**
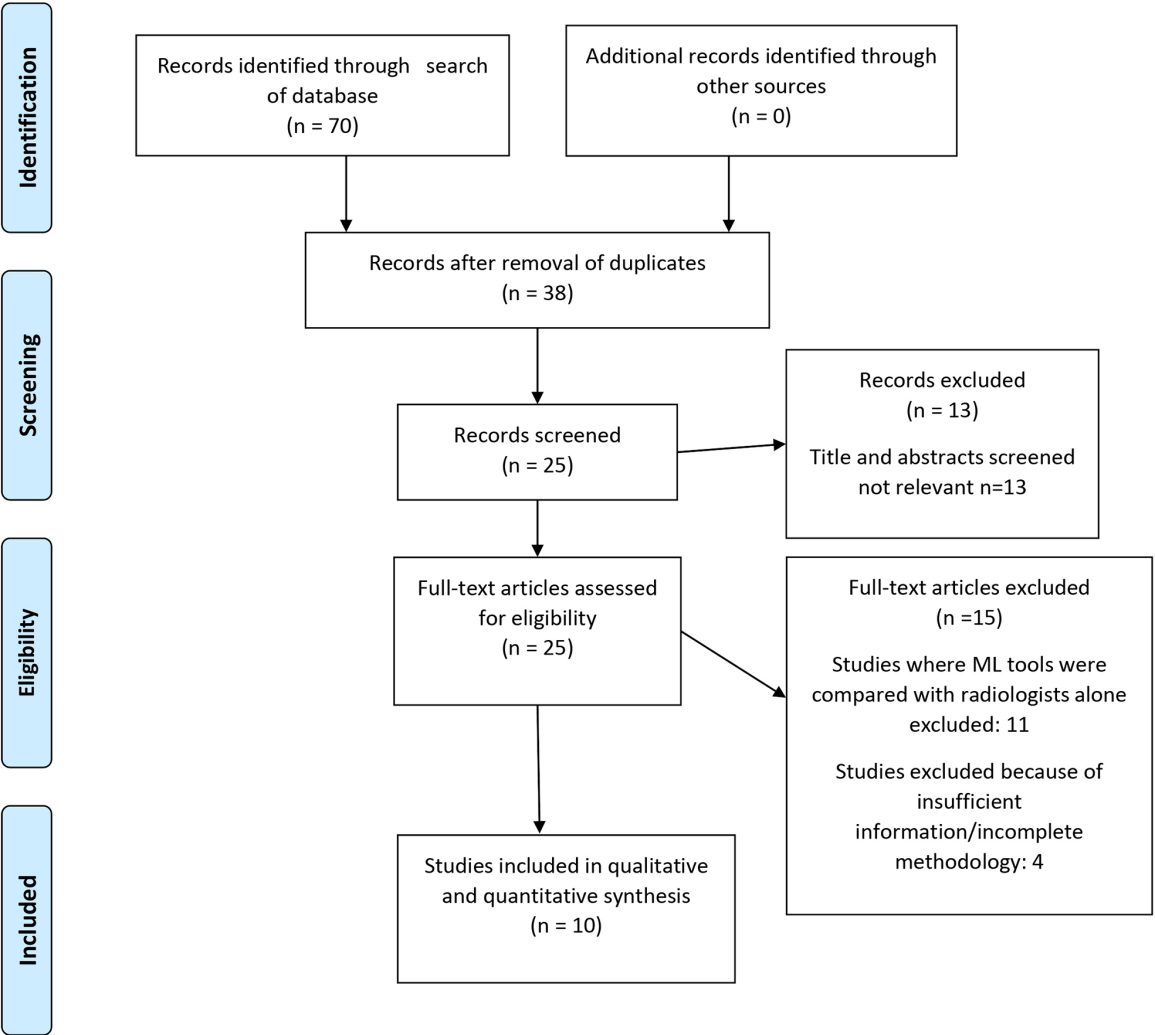
PRISMA 2009 Flow Diagram.

**Figure 2:**
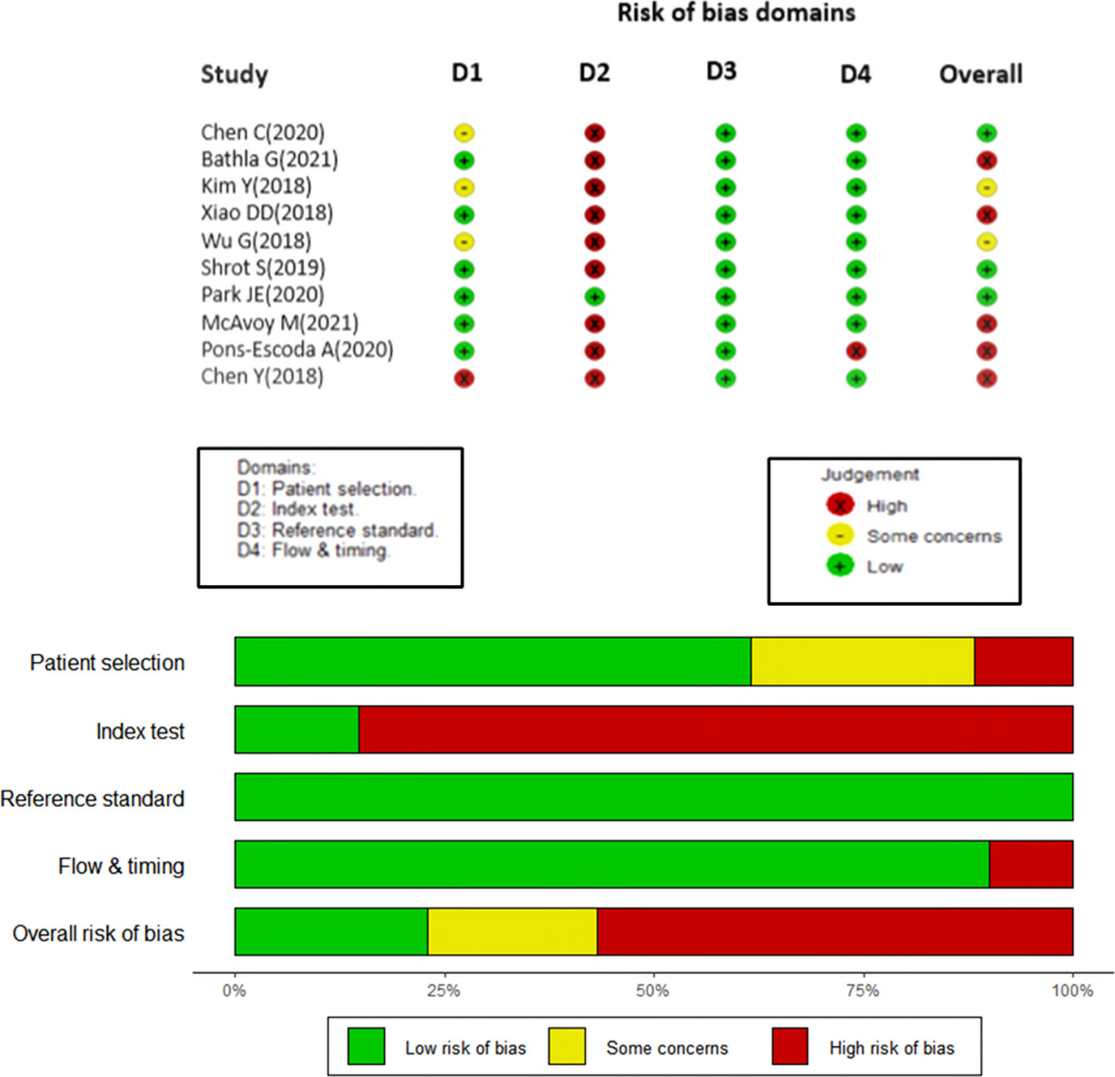
Studies included in the meta-analysis with the quality of diagnostic accuracy studies (QUADAS) scores.

This study was conducted in concordance with Preferred Reporting Items for Systematic Reviews and Meta-Analysis (PRISMA) guidelines ( **Figure 1**). Quality of primary studies was assessed using the QUADAS 2 tool (**Figure 2**).

Literature Search Strategy:

Eligible studies reporting on the diagnostic yield of machine learning or big data in differentiating PCNSL from GBM were identified through a systematic search of the medical literature using a validated search strategy. An electronic search of Medline *via* PubMed, EMBASE and Cochrane database was conducted without any language restrictions from January 1990 till December 2021 to identify potentially relevant articles. Different key-words including Medical Subject Heading (MeSH) terms were combined using Boolean operations ‘AND’ and ‘OR,’ namely, “Magnetic Resonance Imaging” [MeSH] OR “MRI” AND “primary central nervous system lymphoma” [MeSH] OR “brain lymphoma” OR “PCNSL” AND “diagnosis” OR “accuracy” OR “yield” AND “radiomics” OR “Machine learning” OR “deep learning” OR “Artificial Intelligence” OR “AI.” The Cochrane Central Register of Controlled Trials (CENTRAL) and Database of Abstracts of Reviews of Effectiveness (DARE) were also searched electronically from inception until December 2021. Electronic search was further supplemented by hand-searching of review articles, cross references, and conference proceedings.

### Eligibility criteria

#### a. Selection of studies

All studies reporting on ML algorithms that aimed to differentiate between GBM and PCNSL on MR imaging were included. Studies that compared ML with radiologists were excluded in this meta-analysis in order to maintain homogeneity, and we intend to explore this in a subsequent paper. Articles were also excluded if they were commentaries, editorials, letters, or case reports Two reviewers (AG and JSG) extracted relevant data from each selected article, including study characteristics and findings of test results using a standardized data extraction sheet that was verified independently by the third reviewer (A.M). Any discrepancy was resolved by consensus. Quality of individual primary study in the meta-synthesis was assessed using the QUADAS 2 quality assessment tool for studies that uses criteria scored as ‘yes, unclear, or no’ risk of bias, and assigns overall quality rating as ‘low, high, or unclear’ to each individual study. Furthermore, we also used the Radiomic Quality score (RQS), a quality assessment tool specifically developed to evaluate quality of radiomics in neuro-oncology studies (20). Studies were scored upto a maximum of 36, involving six key domains.

#### b. Type of participants

Patients of PCNSL & GBM with pathological confirmation of disease. In addition, all the patients had DICOM MR images of the tumour.

#### c. Diagnostic metrics

The diagnostic metrics included the Sensitivities and specificities of all the included studies. If papers described performance using receiver operating characteristic curves, we back-calculated possible sensitivities and specificities.

### Quality assessment

Quality of primary studies was assessed using the QUADAS 2 tool and the Radiomic Quality Score (RQS) by two independent reviewers [AG and JSG]. The QUADAS-2 tool is recommended by the agency for healthcare research and Quality, the Cochrane Collaboration and the United Kingdom National Institute for Health and Clinical excellence in order to assess the risk of bias among 4 domains (patient selection, index test, reference standard and flow & timing). Any disagreement between the two reviewers were solved by mutual consensus, and then independently scored by a third reviewer (AD). Four main domains including patient selection, index test, reference standard, and flow and timing were evaluated and plotted for various risk bias domains **(** **Figure 2** **)**.

### Statistical analyses

We performed a meta-analysis of the performance of ML and DL algorithms in differentiating PCNSL from GBM. Reference standard was pathologic confirmation on biopsy of concomitant primary CNS lymphoma or GBM. Results for studies pooled in the quantitative analysis were calculated as proportions, with meta-analysis performed using the generalized linear mixed model (random-effects model) to produce summary estimates with 95% confidence intervals (CIs).All statistical analyses were performed on R Studio version 3.6.1.The ‘meta’, ‘mada’ and R-packages were used to draw forest plots. We also used the mada package, a freely available package to construct hierarchical summary receiver operating characteristic (HSROC) models, as recommended by the Cochrane Collaboration for meta-analyses of diagnostic tests. The ‘robvis’ package was used for QUADAS analysis. Balanced accuracy was also calculated using the average of sensitivity and specificity for all the studies. The I (2) was estimated to test level of heterogeneity.

## Results

### Literature search

Seventy studies of interest were found, of which 38 were duplicates. Of the remaining 32, seven were rejected based on title and abstract. Of the twenty-five full-text manuscripts retrieved, ten were selected for this meta-analysis after considering the inclusion and exclusion criteria **(** **Figure 1** **)**. The total sample size in the 10 studies was 1311 and the overall accuracy (9 studies), sensitivity, and specificity values of each study was documented. There was no available accuracy value in one of the studies (21), nor were we able to reverse calculate it with the given information. 5 studies used a 3T MRI scanner, while 3 studies used both 3T and 1.5 T. Two studies (22, 23) did not provide details on the scanner used or the scanning protocol. None of the studies had a prospective design.

All eligible studies were relatively recent, and conducted between 2018 to 2021. 60% of the studies were conducted from hospitals in Asia (China/South Korea). The metananalysis included ten studies that compared PCNSL from GBM. A summary of the general characteristics of included studies is presented in **Table 1**, while the method-related information is summarized in **Table 2**. All studies reported at least one of the following: accuracy, sensitivity, specificity, or AUC **(** **Table 3** **).** Half of the studies used SVM as part of their ML algorithm, while 40% used CNN (23) (23–25), and one paper (26) used step wise selection with unsupervised learning. All of the studies performed some version of internal independent or internal cross-validation to train their ML algorithms, but only one of the studies externally validated their model (24).

**Table 1:** Summary of the study profile & methodology of the reviewed studies.

Sr. No	Author/Year/country	Patient cohort		Classifier/algorithm used	Internal validation set	External validation set	MRI used for Imaging	MRI sequences	Image Segmentation & Feature extraction tool	Reference Standard test	Type of study
		PCNSL	GBM								
1	Chen (1) et al/China/2018	30	66	Convolutional neural network,	Yes, cross validation	Yes	3 Tesla	T1 contrast enhanced	Scale Invariant Feature Transformation (SIFT)	Histopathology	Retrospective study
2	Xiao (2) et al/2018/China	22	60	Machine learning: Naïve Bayes (NB), SVM, LR, Random Forest model	Yes ,10-fold internal cross validation	No	1.5 & 3 Tesla	T1W, T2W & T1W contrast enhanced	Pyradiomics	Histopathology	Retrospective study
3	Guoqing (3) et al/2018/ china	32	70	Convolutional neural network	Yes, Independent set	No	3 Tesla	Not reported	Patch based Sparse representation method	Histopathology	Retrospective study
4	Kim (4) et al/2018/ S Korea	65	78	Logistic regression, SVM, Random Forest model	No	Yes, Independent set	3 Tesla	T1W contrast, DWI, T2W	Pyradiomics	Histopathology	Retrospective study
5	Shrot (5) et al/2019/ Israel	12	41	Machine learning: Binary SVM	Yes, Leave one out cross validation	No	1.5 & 3 Tesla	MR perfusion using DSC images & DTI	3D slicer, intensity-based feature extraction from MR maps	Histopathology	Single Institutional retrospective study
6	Chen (6) et al/2020/ China	62	76	**5 selections**: Distance correlation, RF, LASSO, XG boost, GBDT; **3 Classifiers:** LDA, SVM, LR	Yes, Independent set	No	3 Tesla	T1W contrast	lifeX	Histopathology	Single Institutional retrospective study
7	Park (7) et al/Korea/2020	95	165	Convolutional Neural network	Yes, Internal Validation set	Yes, Independent set	3 Tesla	T1W contrast, T2 FLAIR, DSC	Segmentation done semiautomatically by two neuroradiologists	Histopathology	Retrospective study
8	Escoda (8) et al /2020/Spain	47	48	Logistic binary regression	Yes, Independent set	No	1.5 & 3 Tesla	T1 Contrast, DSC-PWI	3D Slicer, Time intensity curve normalization	Histopathology	Retrospective study
9	Bathla (9) et al /2021/USA	34	60	Machine learning SVM with Polynomial kernel, SVM with radial kernel ,neural network, MLP, Random Forest Model, GBRM, Adaboost	Yes (5 fold cross validation)	No	Not Reported	T1 W contrast, & FLAIR, ADC	Pyradiomics	Histopathology	Single Institutional retrospective study
10	McAvoy (10) et al/2021/USA	135	113	Convolutional Neural network	yes, independent	No	3 Tesla	T1 Contrast	Not Reported	Histopathology	Single Institutional retrospective study

FLAIR, Fluid Attenuation & Recovery; MLP, Multilayer Perception; SVM, Support Vector Machine; GBRM, Generalised Boosted regression Model; DTI, Diffusion tensor Imaging; DSC, Dynamic Susceptibility; ML , Machine learning; CNN, convolutional Neural network; RF, Random forest; LASSO, Least Absolute Shrinkage and Selection Operator; PCA, Principal Component Analysis; LR, Logistic Regression; SVM, Support Vector Machine; LOGISMOS, Layered Optimal Graph Image Segmentation for Multiple Objects and Services; MLP, Multilayer perceptron; SIFT, Scale invariant feature transform; mRMR, Minimum redundancy maximum relevance; CNN, Convolutional neural network; LOOCV, Leave One Out Cross Validation.

**Table 2:** Summary of the results of the reviewed studies.

Sr. No.	Author/Year/country	Diagnostic metrics of the best performing model from the validation set				Study limitations reported	Study strengths reported
		Accuracy	Sensitivity	Specificity	AUC		
1	Chen (1) et al/2018/China	0.906	0.8	0.955	0.982	- Single MR sequence was used	Calculation methods are fast
2	Xiao (2) et al/2018/ China	0.82	0.78	0.91	0.9	- non enhancing & multiple lesions were excluded - Different scanners were used for image acquisition resulting in imaging protocol heterogeinity	- Image pre-processing technique used
3	Guoqing (3) et al/2018/China	0.945	0.9	0.96	NA	Not reported	-Completely automated
4	Kim (4) et al/2018/ S Korea	0.947	0.966	0.929	0.956	- Retrospective study with patient selection bias - MR images of validation & discovery cohort were obtained from the same machine thereby may not be generalizable to other MR machines -Features were chosen empirically	Not reported
5	Shrot (5) et al/2019/Israel	NA	1.00	1.00	NA	-ROI tracing was done manually leading to intra & interobserver variability -Small sample size - Non enhancing part of the tumour was excluded - Impact of each MR sequence on the classification model not reported	Not reported
6	Chen (6) et al/2020/ china	0.979	0.982	0.976	0.978	-Isolated evaluation of T1C images -Diagnostic Performance of radiomics based machine learning was not compared with other MR technology -Small sample size -No external validation	Not reported
7	Park (7) et al/Korea/2020	NA	0.95	0.76	0.89	-Diagnostic performance dropped in external data set due to overfitting - Spatial heterogeneity - Differences in contrast preloading & Image acquisition protocol results in variability of time signal intensity curves	Not reported
8	Escoda (8) et al/2020/Spain	0.93	0.93	0.92	NA	-Retrospective nature of the study. - Wide range of MR sequences	-Near Homogenous Imaging protocol. - Balancing of tumour types - semi-automation in image segmentation & co- registration - Objective approach to classification process
9	Bathla (9) et al /2021/USA	0.934	0.97	0.871	0.977	Small sample size -Absence of external validation set -Did not assess deep neural networks	-Well documented Imaging protocol - use of feature selection techniques, discrimination and nested cross validation
10	McAvoy (10) et al/2021/USA	0.93	1	0.86	0.94 (GBM)	- Retrospective study with small number of patients - Loss of data while exporting the image data sets	Not reported
		0.94	0.87	1	0.95(PCNSL)	

**Table 3:** Summary of the diagnostic metrics of all the studies included in the meta-analysis.

**Author**	**Year**	**Sample Size (N)**	**Accuracy (%)**	**Sensitivity (%)**	**Specificity (%)**	**Balanced Accuracy (%)**	**AUC (%)**
Chen Y (1)	2018	96	90.6	80.0	95.5	87.8	98.2
Xiao DD (2)	2018	82	82.0	78.0	91.0	84.5	90.0
Wu G (3)	2018	102	94.5	90.0	96.0	93.0	NA
Shrot S (5)	2019	53	93.6	100	100	100	NA
Chen C (6)	2020	138	97.9	98.2	97.6	97.9	97.8
Park JE (7)	2020	260	NA	95.0	76.0	85.5	89.0
Escoda A (8)	2020	95	93.0	93.0	92.0	92.5	NA
Bathla G (9)	2021	94	93.4	97.0	87.1	92.1	97.7
McAvoy (10) M	2021	248	94.0	87.0	100	93.5	95.0
Kim Y (4)	2018	143	94.7	96.6	92.9	94.7	95.6

Among 10 studies, seven studies were from single centre, and 3 studies were from multicentre data source (25–27).

### Risk of bias assessment

The QUADAS tool assessment of risk of bias in the included studies are shown in **Figure 2**. In domain 1, 60% studies reported well-documented image acquisition protocols or use of publicly available image databases, with one study having a high risk of bias and two others with unclear risk in accruing for patient selection. The patient selection for these trials was based on a case-control design because outcomes were known prior to implementation of ML.

Additionally, in the second domain (“index tests”), the study designs for the papers examined had prior knowledge of the reference standard prior to implementing the index test, which introduces a high risk of bias. Hence, the authors decided to evaluate only the results of validation/test data set to conduct the statistical analysis in this study. Only one study was externally validated (21), therefore, all the other included studies were assigned a high risk of bias. As noted previously, future studies of ML should attempt to remove this risk of bias as much as possible, ideally by utilizing a prospective design and external validation.

As judged in domain 3, the reference standard of histological diagnosis was considered to provide an accurate classification of the target condition, although this reporting could be improved if the authors provided details regarding how the histological samples were obtained and processed and the specific histological characteristics that determined the diagnosis.

Finally, most of the studies apparently included all eligible patients in the analysis and had clearly defined inclusion and exclusion criteria, with a resultant low amount of bias in the fourth domain, “flow and timing”.

Overall, a high risk of bias was estimated in the studies as summarized in **Figure 2**. Consequently, the quality assessment was limited regarding the applicability of ML based radiomics analysis.

### Assessment of the radiomics quality score

The median RQS score of the 10 studies was 16.0, which was 44.4% of the ideal score of 36 **(** **Table 4** **).** The lowest score was 13 and the highest score was 18 (50% of the ideal quality score). Compared with the ideal score, the RQS of the selected studies was lowest in the high level of evidence domain and open science and data domain (0%), followed by biological/clinical validation, and feature reproducibility in image and segmentation.

**Table 4:** Summary of Radiomics Quality Score (RQS) of individual studies.

Sr. No	Name	RQS score	%	RQS checkpoint 1 (image protocol quality)	RQS checkpoint 12	RQS checkpoint 3
1	Chen Y (1) (2018)	16	44.44%	1	1	14
2	Xiao DD (2) (2018)	16	44.44%	1	1	14
3	Wu G (3) (2018)	15	41.67%	1	1	13
4	Short S (5) (2019)	13	36.11%	1	1	11
5	Chen C (6) (2020)	17	47.22%	1	1	15
6	Park JE (7) (2020)	18	50.00%	1	1	16
7	Pons-Escoda A (8) (2020)	16	44.44%	1	1	14
8	Bathla G (9) (2021)	16	44.44%	1	1	14
9	McAvoy (10) M (2021)	16	44.44%	1	1	14
10	Kim Y (4) (2018)	17	47.22%	1	1	15

Feature reduction was missing from the study with the lowest score (28). Meanwhile, studies with the highest score earned additional points by using validation based on a dataset from another institute.

## Subgroup analysis

### Data extraction

Two of the ten studies (29) (30), utilized a single MRI sequence acquired by either conventional imaging, while the remaining studies implemented both conventional and advanced perfusion and Diffusion Weightage Imaging (DWI) sequences. An imbalance in the ratio of sample size between PCNSL cohort and GBM cohort was observed in all the studies with a ratio of almost 2:1 and 3:1 in favour of GBM cohort. However, two of the studies had a balanced sample size between PCNSL and GBM cohort (27, 29).

### Heterogeneity assessment

Significant heterogeneity was present amongst the included studies regarding their scanning protocols, image sequences selected for analysis, methods of drawing ROI, feature engineering, and methodology of using ML/DL algorithms. The forest plots for balanced accuracy, sensitivity, and specificity were plotted based on the total sample size, and the forest plot for accuracy was plotted based on 1051 samples (excluding the study conducted by Park JE (21) as accuracy data was not available). The I (2) was estimated to test the level of heterogeneity; and since this was greater than 50%, random effect model for meta-analysis was used.

**Figure 3:**
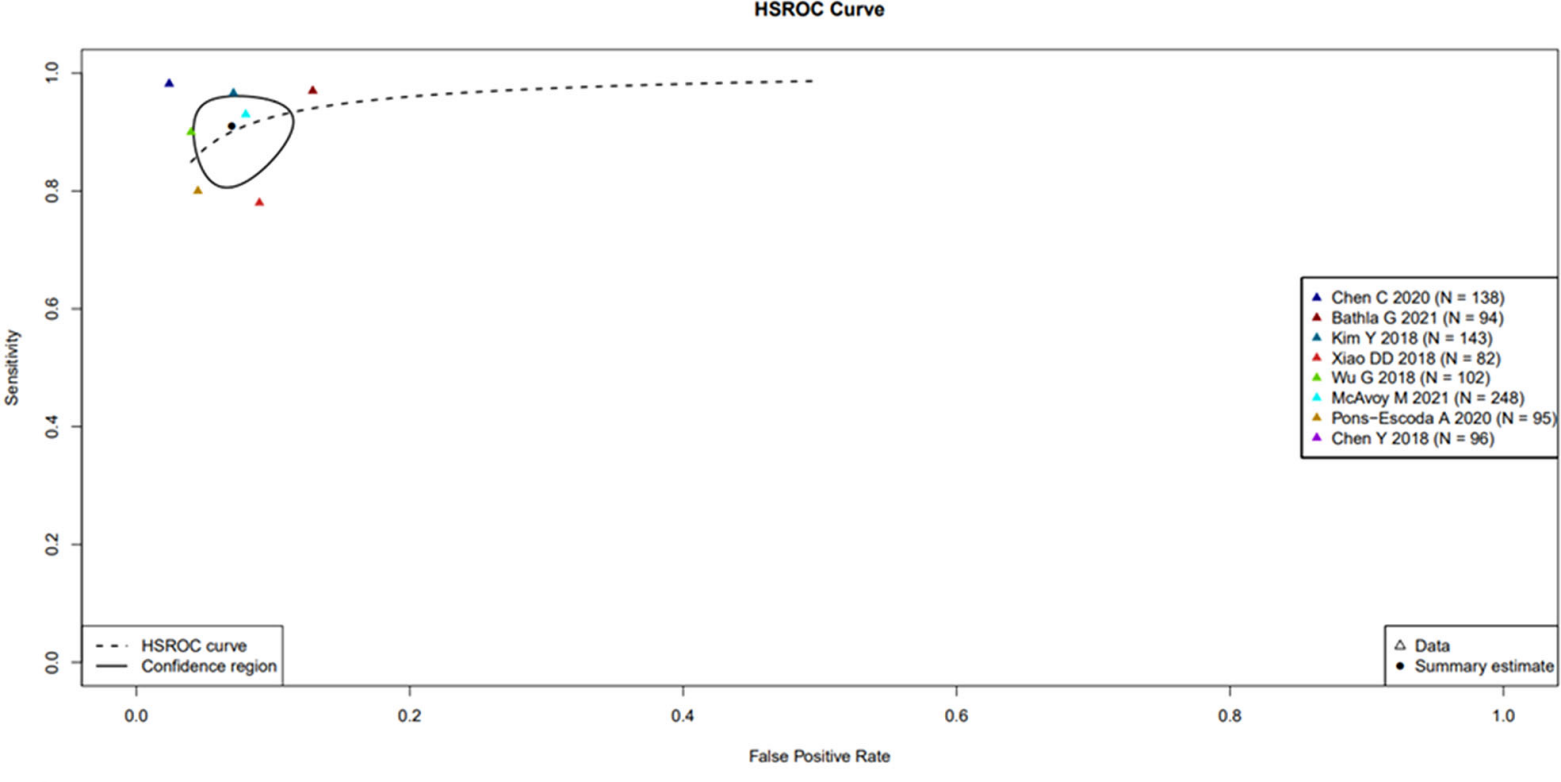
Hierarchical Summary Receiver Operator Curve (HSROC) plot displaying the diagnostic performance of radiomic based ML tools & DL tools in differentiating PCNSL from GBM. Hierarchical Summary Receiver Operator Curve (HSROC) plot displaying the diagnostic performance of radiomic based ML tools & DL tools in differentiating PCNSL from GBM. Each coloured triangle represents each of the studies in the meta-analysis. The plotted curve is the regression line that summarizes the overall diagnostic accuracy. The pooled sensitivity and specificity estimate is based on the assumption of conditional independence and the use of perfect reference standards. The “TP”, “FP”, “FN”, “TN” rates for the two studies (Park JE 2020 and Shrot S 2019 studies) as the former study has no available accuracy value and the latter one has both sensitivity and specificity equal to one.

**Figure 4:**
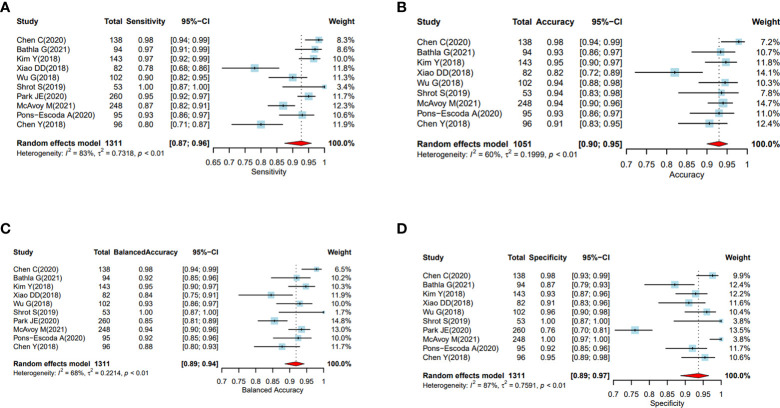
**(A–D)** Performance evaluation of the ML and DL algorithms of all the studies in distinguishing PCNSL from GBM as represented by the random forest plots. **(A)** Forest plots of sensitivity. **(B)**: Forest plots of accuracy. **(C)** Forest plot of balanced accuracy, **(D)** Forest plots of specificity.

A large difference between the confidence region and 95% prediction regions in the Hierarchical Summary Receiver Operator Curve (HSROC) plot curve represents the heterogeneity across the studies in **Figure 3**. A forest plot was drawn to estimate the heterogeneity in sensitivity, specificity, accuracy and balanced accuracy as represented in **Figures 4A–D**. Significant heterogeneity was found in both sensitivity (I (2) 83%, p < 0.01), specificity (I (2) 87%, p ≤ 0.01) and accuracy (I (2) 65%, p ≤ 0.01).

### Threshold effect assessment (HSROC)

The Spearman correlation coefficient between the sensitivity and false-positive rate was − 0.16 (p = 0.66), indicating the absence of a threshold effect. A threshold effect indicates a positive correlation between sensitivities and the false-positive rate that leads to a “shoulder arm” plot in the summary receiver-operating characteristic curve space. However, the visual assessment of the HSROC indicates the absence of a threshold effect as shoulder is absent in the HSROC space.

### Data analysis

The HSROC based on a random effect model was applied to account for both intra- and interstudy variances in analysing the diagnostic accuracy of the ML and DL algorithms utilizing radiomic features for classifying PCNSL from GBM. The area under the curve (AUC) data available from 7 studies showed a ranged between 0.89 to 0.98 in the validation data set indicating high diagnostic performance.

### Subgroup analysis

The pooled sensitivity, specificity, and accuracy were combined using a random effects model because of the heterogeneity across the reviewed studies in **Figures 4A–D**.

In the subgroup analysis, the overall sensitivity of diagnosing PCNSL was lower (92% (95% CI, 0.88, 0.95)) than the specificity (94% (95% CI, 0.89, 0.97)). We did not find any significant differences in sensitivity, specificity or accuracy based on sample sizes less than or greater than 100.

## Discussion

This systematic review and meta-analysis evaluated the efficacy of deep learning/machine learning based algorithms in differentiating PCNSL from GBMs, a dilemma often encountered with neuroradiologists and the neurosurgeons, often requiring invasive biopsies to classify the above entities. Apart from the dilemma of differentiating the above malignant lesions, neuroradiologists often also have difficulty in differentiating PCNSL from inflammatory conditions (multiple sclerosis and tumifactive demyelination) often having therapeutic implications. Therefore, having non-invasive tools like radiomics and AI would help increase the diagnostic ability of the neuroradiologists to differentiate not only malignant from benign inflammatory conditions but also classify malignant lesions like PCNSL and GBM which are hitherto difficult to distinguish using radiological semantic features. This could be further useful in patients where histopathological examination cannot be done due to a multitude of reasons such as deep location within the brain and poor performance status.IN such a scenario, non- invasive methods like radiomics and deep learning from the MR images may help the clinician.

Although radiomics and deep learning algorithms have been used for a multitude of neurological conditions (31–34) its use in classifying malignant conditions and differentiating them is of paramount importance as the therapy and prognosis changes across the spectrum of brain tumours. The present study highlights the use of ML and DL algorithms for discriminating PCNSL and GBM on radiological imaging. We identified 10 studies that trained predictive models using ML or DL algorithms to classify PCNSL from GBM against the reference gold standard histopathology. All the studies, used classifiers that trained on radiomic features extracted from MR images or classifiers using deep learning algorithms like convolutional neural network (CNN).

The pooled analysis of all the studies showed encouraging results with ML or DL classifiers performing extremely well with highest accuracy of 97.9% and the lowest of 82% in differentiating PCNSL from GBM. The area under the curve (AUC) ranged between 89%-98.2% among all the studies that were reviewed. (**Table 4** and **Figure 4**)

The diagnostic metrics from the pooled analysis of the results of the 10 studies showed a high degree of concordance in classifying PCNSL from GBM as against the reference histopathology. However, these positive results must be interpreted with caution as a multitude of factors such as small sample size, heterogenous imaging protocols, patient selection criteria into the training and the validation set may have led to overfitting of the data at the time of model development. Overfitting is common in radiomic studies involving machine learning and deep earning classifiers that reduces its potential for immediate incorporation into clinical practise and use it for treatment decisions (29–31).

Therefore, ML and DL classifiers need to be trained in large data sets using highly heterogenous population. Further, these models (classifiers) show variations with subtle changes in the methods of segmentation, pre-processing of MR images acquired from heterogenous MR machines. A previously conducted systematic review and meta-analysis in 2018 included 8 studies which used ML based classifiers for differentiating PCNSL from GBM. Seven of the eight studies did not have any external validation except for one study in which the ML classifier modelled on the training set was validated on an external data set (32). Similar to the above metaanalysis, our metaanalysis also had a single study that was externally validated on a different data set. There has been a spurt in publications on ML/DL models in classifying PCNSL versus GBM since 2018, and we found a total of 24 articles investigating role of ML and DL algorithms for classifying PCNSL and GBM. In order to make the meta-analysis more robust, we focussed on studies reporting on the performance of their ML/DL models exclusively against the reference standard (histopathology).

A recent systematic review from 23 studies also investigated the role of DL & ML in differentiating PCNSL from all grades (Grade-II-IV) of Gliomas (31). However, significant differences exist in the methodology and the search strategy of our metaanalysis. Moreover, our metaanalysis included only those studies that used ML or AL for differentiating PCNSL from Glioblastoma against the gold standard histopathology. By combining all the studies, on DL/ML in differentiate ng PCNSL from GBM, they were left with a heterogenous dataset precluding any further mathematical analysis to derive a meaningful data, and hence had to contend with only a systematic review of the available literature.

However, at the very outset and literature search strategy stage of our manuscript, we identified the heterogeneity in methodology of the conducted studies, and realised they could be broadly classified into 2 types- those comparing ML/DL with histopathology as a gold standard, and then those comparing ML/DL models with radiologists performance. We found around 12 papers under each category, and analysed them separately. This current manuscript deals with the performance of ML/DL methods versus histopathology as a gold standard. Hence, mathematical analysis in the form of statistical tests for a meta-analysis were performed to evaluate the proof of performance of advanced computing methods in differentiating PCNSL from GBM and not other gliomas.

To summarise, ML and DL tools may complement the radiologic features to differentiate PCNSL from GBM. These tools may have the potential to assist radiologists in approaching cases that may have features common to both PCNSL and GBM. Presently these algorithms may have certain deficiencies, however with refinement in the computing processes, ML/DL based models will likely help the neurosurgeons improve the quality of managing patients of brain tumours by optimizing the use of invasive diagnostic procedures in the future, thereby reducing the incidence of complications that compromise patient quality of life and life expectancy while expediting initiation of intervention.

Strengths of the study

We assessed ML and DL performance in both internal and external validation data sets which enhanced the credibility of the review. Being able to compare both the analytic methods to the gold standard histopathology in the test cohort and validation cohort have produced fairly clear results.

Limitations of the Study

Application of ML in neuroradiology for solving the dilemma of whether an image depicts GBM or PCNSL is relatively new. There is currently a limited number of publications that address this scientific inquiry. Our search strategy for the present study only included limited databases (PubMed, EMBASE & Cochrane database). All the studies that were reviewed varied in terms of the imaging protocols used, types of MRI machines used, MR sequences used (i.e., T1-weighted, T2-weighted, diffusion-weighted, etc.), method of tumour segmentation, tools for feature selection and reduction and ultimately the types of classifiers used for training the image datasets. Future studies that address distinguishing GBM from PCNSL should prospectively evaluate the performance of their model and also consider the utility of newer MRI techniques that may improve differentiation of these two pathologies. Additionally, our assessment of bias revealed inherent issues with applying the QUADAS-2 to ML studies. Despite these limitations, we maintain that assessment of bias is an absolute necessity.

### Future directions:

Prospective multicentre trials are the need of the hour to generate more robust data so that results from an independent external validation dataset are available. The inherent variability across studies with regard to the process of conducting each step leading to the radiomics model could be attributed to high bias and heterogeneity, not necessarily underlying biologic effects, standardization in image acquisition, segmentation methodology, feature selection and classification, statistical analysis, and the reporting format should be established for reproducibility and the generalization of ML-based radiomics studies (33). Essential steps for standardization include optimizing the standard imaging acquisition process, fully automating the process for segmentation and feature engineering, reducing the redundancy of feature numbers, enhancing the reproducibility of radiomics features, and reporting the results transparently. The guidelines suggested by the relevant professional societies, such as the Society of Nuclear Medicine and Molecular Imaging, the Quantitative Imaging Network, Radiology Society of North America, and the European Society of Radiology that lead the field in imaging methods, including radiomics, should be considered (34).

## Conclusion

The systematic review of studies investigating ML & DL based algorithms to differentiate PCNSL from GBM have demonstrated encouraging results and certainly have the potential to aid neurooncologists in taking preoperative treatment decisions in the future leading to not only reduction in morbidities but also be cost effective. It is likely that predictive analytics using ML or DL based algorithms will help optimize diagnostic decision-making process and individualise patient management. Although studies had limited sample size, formal predictive analytics, using these models may have the potential to improve clinician performance complementing human expertise and experience with the computational power. However, one must keep in mind the pitfalls associated with overfitting the data due to limited image data sets and resultant lack of training these algorithms to maximize the generalizability and their utility. Therefore, prospective multicentric trials with large data sets should be initiated to train the models on large heterogeneous and real-world data sets that account for the heterogeneity encountered in acquisition of images in the real-world clinical practice.

## Data availability statement

The original contributions presented in the study are included in the article/supplementary material. Further inquiries can be directed to the corresponding authors.

## Author contributions

AG & JG: Conceptualisation and designing the study, writing the manuscript, analysing the results. ST, SH, JG: statistical analysis. AD: verification of results, bias risk assessment, AM: review of manuscript. All authors contributed to the article and approved the submitted version.

## Conflict of interest

The authors declare that the research was conducted in the absence of any commercial or financial relationships that could be construed as a potential conflict of interest.

## Publisher’s note

All claims expressed in this article are solely those of the authors and do not necessarily represent those of their affiliated organizations, or those of the publisher, the editors and the reviewers. Any product that may be evaluated in this article, or claim that may be made by its manufacturer, is not guaranteed or endorsed by the publisher.
